# Human Embryonic Stem Cell Lines with Lesions in *FOXP3* and *NF1*

**DOI:** 10.1371/journal.pone.0151836

**Published:** 2016-03-18

**Authors:** Hui Zhu, Barry Behr, Vikrant V. Reddy, Mark Hughes, Yuqiong Pan, Julie Baker

**Affiliations:** 1 Department of Genetics, Stanford University, Stanford, California, 94305, United States of America; 2 Stanford IVF Laboratory, Stanford Fertility and Reproductive Health, Lucile Packard Childrens Hospital Stanford, Palo Alto, California, 94304, United States of America; 3 Department of Obstetrics/Gynecology, Stanford University School of Medicine, Stanford, California, 94305, United States of America; 4 Genesis Genetics Institute, Plymouth, Michigan, 48170, United States of America; 5 Department of Medicine, Division of Blood and Marrow Transplantation, Stanford University, Stanford, California, 94305, United States of America; University of Kansas Medical Center, UNITED STATES

## Abstract

Human embryonic stem cells (hESCs) are derived from the inner cell mass (ICM) of blastocyst staged embryos. Spare blastocyst staged embryos were obtained by in vitro fertilization (IVF) and donated for research purposes. hESCs carrying specific mutations can be used as a powerful cell system in modeling human genetic disorders. We obtained preimplantation genetic diagnosed (PGD) blastocyst staged embryos with genetic mutations that cause human disorders and derived hESCs from these embryos. We applied laser assisted micromanipulation to isolate the inner cell mass from the blastocysts and plated the ICM onto the mouse embryonic fibroblast cells. Two hESC lines with lesions in *FOXP3* and *NF1* were established. Both lines maintain a typical undifferentiated hESCs phenotype and present a normal karyotype. The two lines express a panel of pluripotency markers and have the potential to differentiate to the three germ layers *in vitro* and *in vivo*. The hESC lines with lesions in *FOXP3* and *NF1* are available for the scientific community and may serve as an important resource for research into these disease states.

## Introduction

Human embryonic stem cells (hESCs) carrying genetic mutations offer the potential to model genetic diseases, especially if the affected tissues can be reliably generated by differentiating the cells *in vitro* [1]. Models like hESCs are critical to assess the biochemistry of disease and for pharmaceutical approaches such as screening for drug targets. While genome editing has become more tractable in hESCs [2] and iPSCs are now widely used to examine disease mutations, derivation of hESCs following preimplantation genetic diagnosis (PGD) allows for yet another option toward establishment of human genetic models specific for disease phenotypes [1]. As such, PGD derived hESC lines should be an important aspect of the stem cell repertoire and affected PGD embryos–otherwise discarded–should be banked for derivation and subsequent modeling.

IPEX (immunodysregulation polyendocrinopathy enteropathy X-linked syndrome) is an X-linked syndrome characterized by autoimmune-dysfunction, polyendocrinopathy and enteropathy [3, 4]. IPEX is caused by mutations in *FOXP3*, which encodes a forkhead (FKH) family transcription factor and is located on chromosome Xp11.23 [3–5]. Mutation of *FOXP3* was initially identified as being responsible for an X-linked recessive inflammatory disease in mice and subsequently for IPEX in humans [5–7]. In humans, IPEX syndrome leads to childhood mortality.

Neurofibromatosis type 1 is one of the most frequent genetic diseases (1/2500-1/3000) for which no specific treatment exists [8]. This autosomal dominant disorder affects the nervous system and causes neurofibromas [8]. Neurofibromatosis type 1 is caused by mutations of the *NF1* gene [9, 10], which encodes neurofibromin, a negative regulator of *Ras*, and regulates nerve cell growth and tumorigenesis [11, 12].

In this report, we derive two hESC lines from PGD embryos diagnosed as containing lesions that cause the monogenic diseases IPEX and neurofibromatosis type 1. We used laser-assisted micromanipulation to increase efficiency of derivation for these rare embryos to 30% and find that these hESCs are karyotypically normal, male and contain the identical lesions identified from the PGD analysis. Further, we show that the *FOXP3* mutation is caused by a lack of exon 8, demonstrating a splicing mechanism responsible for the mutation. Overall, we suggest that hESCs derived from PGD embryos are an important and untapped resource for studying human disease.

## Materials and Methods

### Human PGD embryos

Human embryos after PGD cycles were donated by couples undergoing IVF treatment. All PGD embryos were obtained from the RENEW biobank of Stanford University after patient written consent. The study was approved by the panel of Stem Cell Research Oversight (SCRO) and the panel of Institutional Review Board (IRB) for Human Subjects Research at Stanford University. PGD procedures were performed on biopsied blastomeres by Genesis Genetics Institute (Detroit, MI). The PGD embryos affected with a *FOXP3* (IVS8+5G>A) mutation and the PGD embryos heterozygous for a *NF1* (IVS1+1G>C) mutation were used for the derivation of hESC lines with lesions in *FOXP3* and *NF1*.

### Derivation of FOXP3-hESC and NF1-hESC lines

PGD embryos were thawed using a Vitrification Warming Kit (Origio, Inc., Trumbull, CT) and transferred into Quinns Advantage Blastocyst Medium supplemented with 20% Quinns Advantage Serum Protein Substitute (Origio, Inc., Trumbull, CT), according to the manufacturer’s instructions. PGD embryos were cultured for one day (S1A Fig) and then the ICM were isolated from expanded blastocysts at day 5 or day 6 post-fertilization by using laser (LYKOS Clinical Laser System, Hamilton Thorne, Beverly, MA) assisted micromanipulation as previously described [13, 14]. Generally, the blastocyst was anchored using a holding pipette and a biopsy micropipette used to remove mural trophectoderm (TE) (S1B Fig). Afterwards, the ICM with a few polar TE cells attached was gently drawn into the biopsy micropipette and released (S1C Fig). If the ICM cells were difficult to separate, the TE cells were hit by laser pulses and aspirated into the holding pipette (S1C Fig).

The isolated ICMs were plated on the Mitomycin-C (Sigma, St Louis, MO) inactivated mouse embryonic fibroblast (MEF) feeder layer cells, and further cultured at 37°C in 5% CO_2_, 5% O_2_ atmosphere (S1D Fig). For the preparation of feeder layer cells, MEF cells were isolated from 12.5 d.p.c. (day post-coitum) embryos of CF1 pregnant mice and cultured for one passage followed by treated with 1 μg/ml Mitomycin-C overnight. The mitotically inactivated MEF cells were cryopreserved in liquid nitrogen. One day before PGD embryo dissection, feeder cells were thawed and plated in 10% gelatin coated 4-well tissue culture dishes (Thermo Fisher Scientific, Inc., Waltham, MA) in DMEM medium supplemented with 10% FBS. Before the isolated ICM was plated, the feeder cell culture medium was changed to derivation medium: half hESC culture medium and half conditional medium (hESC medium conditioned overnight on primary MEF cells). hESC medium comprised DMEM/F12 medium supplemented with 20% KnockOut serum replacement, 0.1 mM nonessential amino acids (NEAA), 1 mM GlutaMAX^TM-1^, 0.1 mM 2-mercaptoethanol (all from Invitrogen, Carlsbad, CA) and 8 ng/ml recombinant human FGF2 (Peprotech, Rocky Hill, NJ). For the first several days of derivation, the medium was changed every other day, and then switched to daily once cells began to expand.

ICMs attached within the first two days after being plated while appearance of cellular outgrowths was variable and occurred even as far out as 20 days. Large cell clusters were dissected mechanically under a microscope using pasteur pipettes that have been pulled into fine needles and blunted over a flame. The dissected cell pieces were transferred using a pasteur pipettes onto plates with fresh feeder cells. During the first several passages of derivation, we mechanically dissected colonies and passaged to fresh feeder cells. Early passages and later passages of hESCs with disease lesions were cryopreserved in freezing medium containing 90% Fetal Bovine Serum (Invitrogen, Carlsbad, CA) and 10% Dimethyl Sulfoxide (Sigma-Aldrich Ltd, Dorset, UK) and stored in liquid nitrogen.

### Characterization of FOXP3-hESC and NF1-hESC lines

#### Immunofluorescence staining

Cells on cover glasses were fixed with 4% paraformaldehyde for 15 min at room temperature. For OCT4 immunostaining, cells were permeabilized with 1% TritonX-100/PBS for 30 min at room temperature. Subsequently, cells were blocked with PBS-BT (1× PBS, 3% BSA, and 0.1% Triton X-100) for 30 min at room temperature. Coverslips were then incubated in primary and secondary antibodies diluted in PBS-BT. OCT4 (sc-9081) antibody was purchased from Santa Cruz (Santa Cruz Biotechnology, Inc.). SSEA4 (MAB4304), SSEA3 (MAB4303), and SSEA1 (MAB4301) antibodies were obtained from Millipore (EMD Millipore Corporation, Billerica, MA). TRA-1-60 (MAB4381) and TRA-1-81 (MAB4381) antibodies were purchased from MILLIPORE. FOXP3 antibody (ab10563) was purchased from Abcam (Abcam Inc., Cambridge, MA). Cell nuclei were stained with DAPI (Sigma-Aldrich Ltd, Dorset, UK). Images were acquired with fluorescence microscope (Leica DC 500).

#### Karyotype analysis

hESCs were passaged onto dishes coated with matrigel (BD Biosciences, San Diego, CA) and cultured in hESC conditional medium. Karyotype was examined on actively divided cells by Cytogenetics Laboratory of Stanford Hospital and Clinics.

#### Analysis of differentiation of hESC lines *in vitro* and *in vivo*

Undifferentiated hESCs growing on feeder cells were passaged onto dishes coated with matrigel (BD Biosciences, San Diego, CA) and cultured in hESC conditional medium. Cell differentiation into endoderm, ectoderm, and mesoderm *in vitro* and the three germ layer population characterization were performed according to Human Pluirpotent Stem Cell Functional Identification Kit (R&D Systems, Inc., Minneapolis, MN). The Endoderm, Ectoderm, or Mesoderm Differentiation Media was prepared by dilution the Endoderm, Ectoderm, or Mesoderm Differentiation Supplement stock solution in Differentiation Base Media, respectively. The Differentiation Base Media was prepared by dilution of 10X Differentiation Base Media Supplement with PPMI and GlutaMAX. All the media supplement reagents are provided in the Pluirpotent Stem Cell Functional Identification Kit. The differentiation toward endoderm, ectoderm, or mesoderm lineage cells was induced by replacing hESC conditional medium with Endoderm, Ectoderm, or Mesoderm Differentiation Media, respectively. After 2–4 days differentiation, the three germ layer lineage markers were characterized by immunofluorescence staining. For embryoid body (EB) differentiation details, see Supplementary Experimental Procedures in S1 File.

Pluripotency of hESCs *in vivo* was analyzed by teratoma formation assay in severe combined immunodeficient (SCID) mice (CBySmn.CB17-Prkdcscid/J #1803, Jackson Laboratory, Bar Harbor, Maine) following approved protocol of Administrative Panel on Laboratory Animal Care (APLAC) of Stanford University. FOXP3-hESC and NF1-hESC cell colonies were dissociated by collagenase and 1X10^6^ cells were suspended in and mixed (1:1) with Matrigel (BD Biosciences) and then injected by using 27GA1/2 needles intramuscularly into the left gastrocnemius muscle of 7~9-week-old mice. After 10–12 weeks, the tumors were removed, fixed in 4% formalin, embedded in paraffin. The histology was analyzed after hematoxylin and eosin staining.

### Genetic testing of FOXP3-hESC and NF1-hESC lines

Genomic DNA was extracted from FOXP3-hESC cells and NF1-hESC cells. Primers for amplifying exon regions of *FOXP3* are listed in Table A in S1 File.

Touch down PCR analysis for IVS8+5G>A in *FOXP3* was performed: 94°C for 5 min followed by 10 cycles consisting of denaturation (95°C, 30 s), annealing (68°C, 45 s), and extension (72°C, 45 s), and then 25 cycles consisting of denaturation (95°C, 30 s), annealing (58°C, 45 s), and extension (72°C, 45 s), with a final incubation at 72°C for 7 min. The PCR reaction includes: 50 ng genomic DNA, 0.4 μM forward and reverse primers, 0.2 mM dNTP. The PCR products were extracted by using MinElute Gel Extraction kit (QIAGEN, Duesseldorf, Germany) followed by DNA sequencing.

Primers for amplifying *FOXP3* cDNA containing exon 6-7-8 and exon 6-7-9 are listed in Table A in S1 File. Primers for amplifying the whole *FOXP3* cDNA are listed in Table A in S1 File. First total RNA was extracted by using RNeasy Plus Mini Kit (QIAGEN, Duesseldorf, Germany). The RNA concentration and purity were measured by NanoDrop (Thermo Scientific, Wilmington, DE) and then used for reverse transcription with random hexamers using SuperScript III First-Strand cDNA synthesis kit (Invitrogen). PCR was performed: 94°C for 5 min followed by 35 cycles consisting of denaturation (95°C, 30 s), annealing (58°C, 30 s), and extension (72°C, 30 s), with a final incubation at 72°C for 10 min. The PCR products were extracted followed by DNA sequencing. The whole FOXP3 cDNA PCR product was extracted and subcloned to pCDH-EF1-T2A-puro expression vector followed by plasmid sequencing.

Primers for the region surrounding IVS1+1G>C in *NF1* gene are listed in Table A in S1 File. Touch down PCR analysis for IVS1+1G>C in *NF1* gene was performed: 94°C for 5 min followed by 10 cycles consisting of denaturation (95°C, 30 s), annealing (65°C, 45 s), and extension (72°C, 45 s), and then 25 cycles consisting of denaturation (95°C, 30 s), annealing (55°C, 45 s), and extension (72°C, 45 s), with a final incubation at 72°C for 7 min. The PCR reaction includes: 50 ng genomic DNA, 0.4 μM forward and reverse primers, 0.2 mM dNTP. The PCR products were extracted followed by DNA sequencing.

## Results and Discussion

### Derivation of FOXP3-hESC and NF1-hESC lines

**Fig 1:**
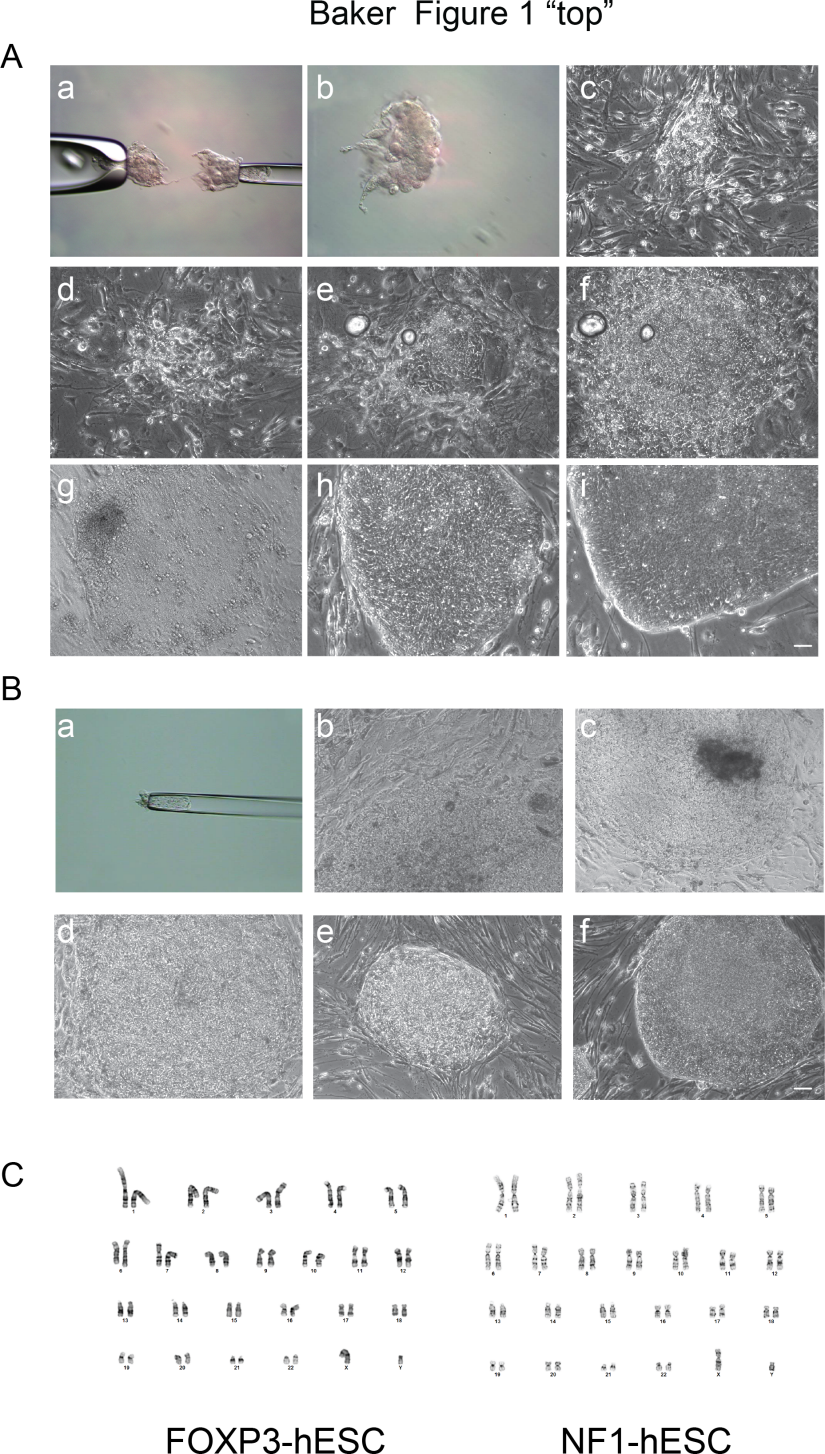
Derivation of FOXP3-hESC and NF1-hESC lines. (A) Representative images of derivation of FOXP3-hESC line. (a) ICM and polar trophectoderm were separated from mural trophectoderm. (b) Isolated ICM. (c) ICM attached to feeder layer two days after plating and cell outgrowth appears. (d) Outgrowth 6 days after plating. (e) Outgrowth 15 days after plating showing small formation of colony. (f) Outgrowth/colony 17 days after plating. (g) 3 days after first passage. (h) Passage 22. (i) Stem cells propagated after thawing and expanding one vial of cryopreserved at passage 12 cells. (B) Representative images of derivation of NF1-hESC line. (a) ICM and polar trophectoderm separated from mural trophectoderm. (b) Cell outgrowth/colony 20 days after plating. (c) 5 days after first passage. (d) 5 days after second passage. (e) Passage 6. (f) Stem cells propagated after thawing and expanding one vial of cryopreserved at passage 7 cells. Scale bars: 50 μm. (C) A representative chromosome spread of FOXP3-hESC (46, XY) and NF1-hESC (46, XY) lines. A total 20 metaphase spreads of each line were examined.

To derive hESCs for use as models of IPEX or Neurofibromatosis type 1, we consented patients to obtain frozen embryos diagnosed with either a lesion in *FOXP3* (four embryos) or *NF1* (seven embryos) following PGD. All embryos were thawed and cultured for one day to reach expanded blastocyst stage at day 5 or day 6 post-fertilization, and transferred to blastocyst medium [15, 16]. After one day in culture, only two blastocysts carrying the *FOXP3* mutation and four blastocysts with the *NF1* mutation survived. As PGD embryos are challenging to process, are limited in quantity, and require freeze-thaw cycle which leads up to 50% lethality, we optimized the derivation protocol by using surgical microdissection to specifically isolate ICM from the mural trophectodermal cells (Fig 1A-a and 1B-a) and then plated the cells on MEF feeder layers (Fig 1A-b).

One ICM from the *FOXP3* donations survived with a cellular outgrowth appearing as early as day 2 post plating (Fig 1A-c) and small colony appearing at day 15 (Fig 1A-e). At day 17, a colony containing a mixture of undifferentiated hESC-like cells and differentiated cells was dissected into 3–4 pieces and transferred to fresh feeder cells (passage 1; Fig 1A-f). Three days after passaging, a flat colony formed with the morphological appearance of hESCs (Fig 1A-g). From passage 2 to 22, robust hESC- like colonies propagated and were easily expanded (Fig 1A-h).

One ICM from the *NF1* donation gave rise to robust cell colony which was ready to passage after 20 days post plating (Fig 1B-b). The colony was dissected into pieces and transferred to fresh feeder cells (passage 1). After passage 6, robust and compact colonies with distinct borders formed and were able to be stably propagated (Fig 1B-e).

Both the FOXP3-hESC and NF1-hESC lines are cryopreserved and survived subsequent thawing and prolonged passaging (Fig 1A-i, thawing of cryopreserved at passage 12 cells; 1B-f, thawing of cryopreserved at passage 7 cells). Established FOXP3-hESC and NF1-hESC lines present typical hESC cellular morphology featured compact dome-like colony structure, distinct colony border and high nucleus to cytoplasm ratio (Fig 1) [17]. Karyotype analysis of both FOXP3-hESC and NF1-hESC lines showed a normal diploid male (46, XY) (Fig 1C) after 20 passages. As the FOXP3 line is male and the disease is X-linked, these cells should be affected for the mutation. The NF1 line should also be affected as mutations in NF1 have a dominant inheritance pattern.

### FOXP3-hESC and NF1-hESC lines are pluripotent

**Fig 2:**
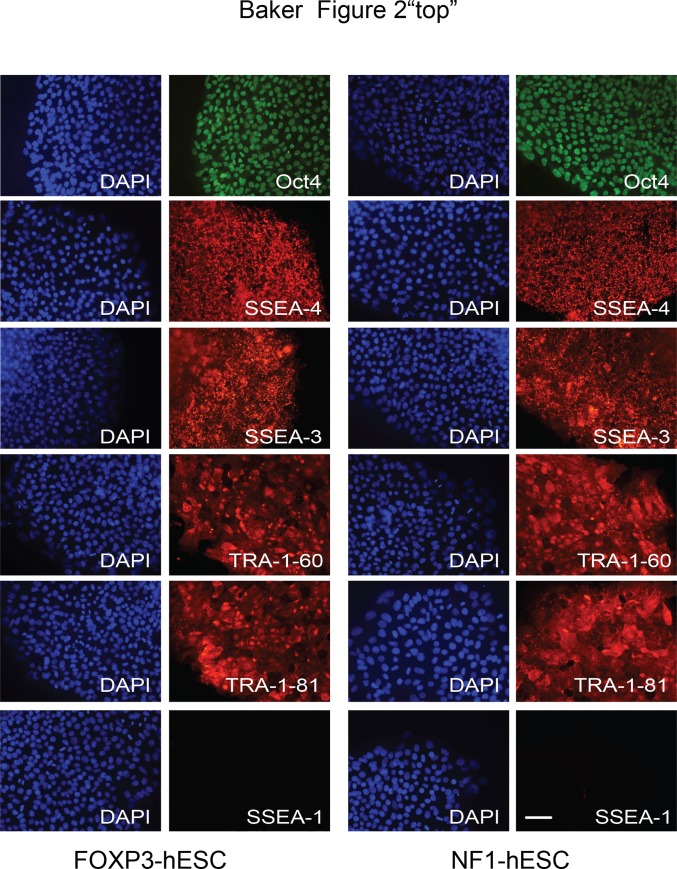
Expression of pluripotent markers in FOXP3-hESC and NF1-hESC lines. DAPI (left panels) and Immunofluorescence staining (right panels) for OCT4, SSEA4, SSEA3, TRA-1-60 and TRA-1-81. SSEA-1 was negative in both cell lines. Left side are images for FOXP3-hESCs and right side are images for NF1-hESC. Scale bars: 50 μm.

To characterize the FOXP3-hESC and NF1-hESC lines, we showed that both cell lines express the pluripotency markers: OCT4, SSEA3, SSEA4, TRA-1-60- and TRA-1-81 (Fig 2). Conversely, we do not detect the expression of SSEA-1, which is consistent with the hESC state (Fig 2) [18]. Gene expression analyses show that both FOXP3-hESC and NF1-hESC lines express pluripotency marker *OCT4*, *SOX2* and *NANOG* at comparable levels as in H9-hESC line (S2 Fig).

**Fig 3:**
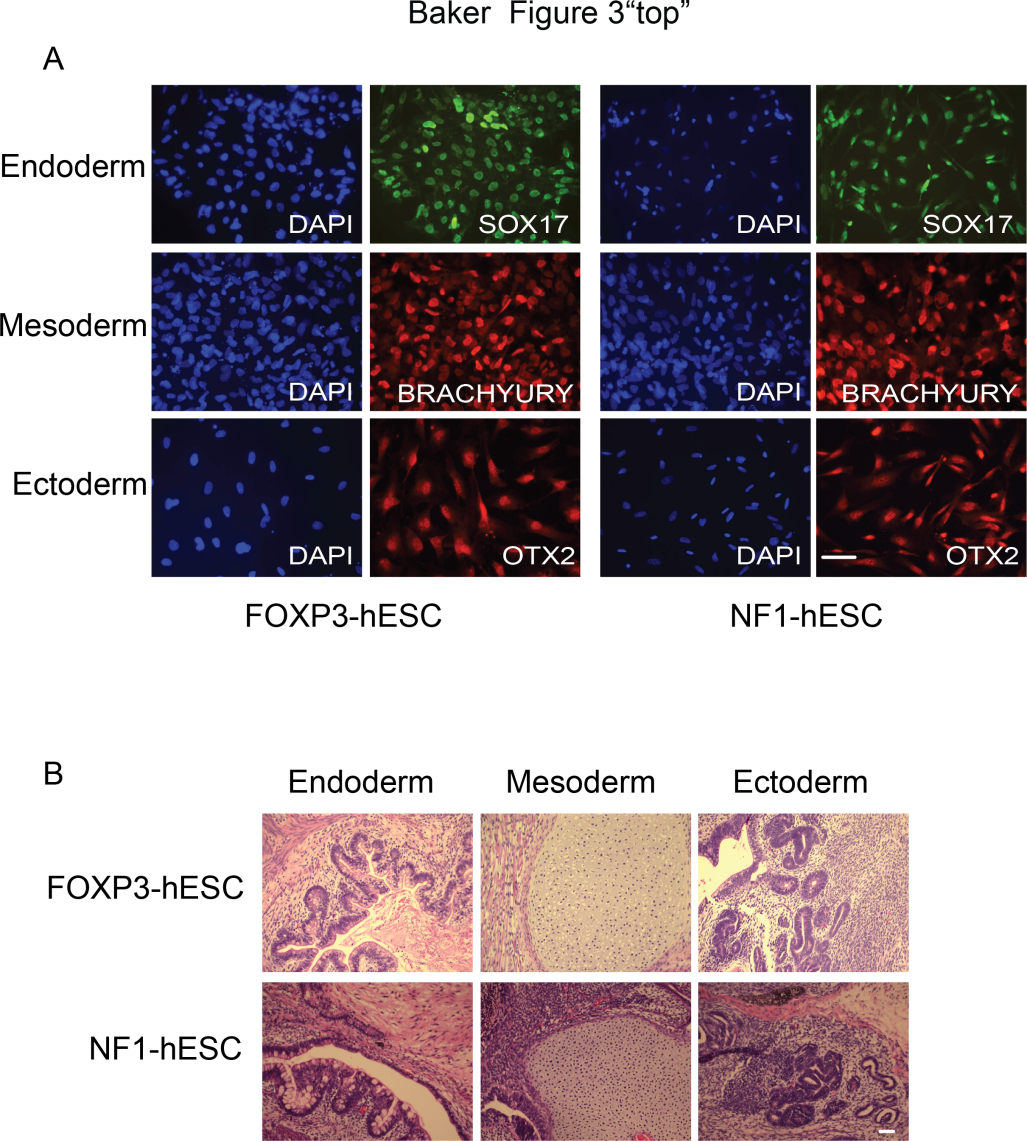
FOXP3-hESC and NF1-hESC lines differentiate into cells of all three germ layers. (A) FOXP3-hESC and NF1-hESC lines were differentiated into endoderm, mesoderm and ectoderm. Immunofluorescence staining showed expression of SOX17, BRACHYURY and OTX2, representing differentiation into endoderm, mesoderm and ectoderm, respectively. Scale bars: 50 μm. (B) FOXP3-hESC and NF1-hESC lines were injected into the gastrocnemius muscle of SCID mice and teratomas developed after 10–12 weeks. Hematoxylin-eosin stained histological sections of the teratoma show glandular epithelia, cartilage and neural rosettes, representing differentiation into endoderm, mesoderm and ectoderm, respectively. Scale bars: 50 μm.

We next tested the ability of FOXP3-hESC and NF1-hESC lines to form all three germ layers using differentiation *in vitro* and teratoma formation. To this end, we differentiated both hESC lines into endoderm, mesoderm or ectoderm and examined these cells for lineage specific markers by immunostaining (see Methods for details). We found that both lines could express markers for endoderm (SOX17), mesoderm (BRACHYURY) and ectoderm (OTX2), strongly suggesting that these lines maintained their developmental potential (Fig 3A). We also differentiated FOXP3-hESC and NF1-hESC lines to embryoid bodies. We show that markers for multiple lineages, including endoderm (*SOX17* and *GATA4*), mesoderm (*BRACHYURY*) and trophectoderm (CDX2 and *CGA*) are upregulated in both lines (S3 and S4 Figs). Ectoderm markers *PAX6* and *NESTIN* are upregulated in FOXP3-hESC differentiated embryoid bodies, however, these two genes are not successfully induced in NF1-hESC lines upon differentiation. Consistent with protein level, another ectoderm marker OTX2 is upregulated in NF1-hESC differentiated embryoid bodies. This result suggests that *NF1* mutation in NF1-hESC line probably affects certain ectoderm, for example, neuroectoderm, lineages differentiation. To test whether these cells could form teratomas containing cells of all three lineages, we next inoculated cell clumps of FOXP3-hESC and NF1-hESC lines to the gastrocnemius muscle of SCID mice. We found that teratomas formed at 10–12 weeks after inoculation. When we examined these teratomas histologically, we found evidence of tissues from all three germ layers: glandular epithelia (endoderm); cartilage (mesoderm); neural rosettes (ectoderm) (Fig 3B). Overall, this strongly suggests that both the FOXP3-hESC and NF1-hESC lines are capable of differentiating into all three germ layers and thus maintain their pluripotent state.

### Genetic testing of FOXP3-hESC and NF1-hESC lines

**Fig 4:**
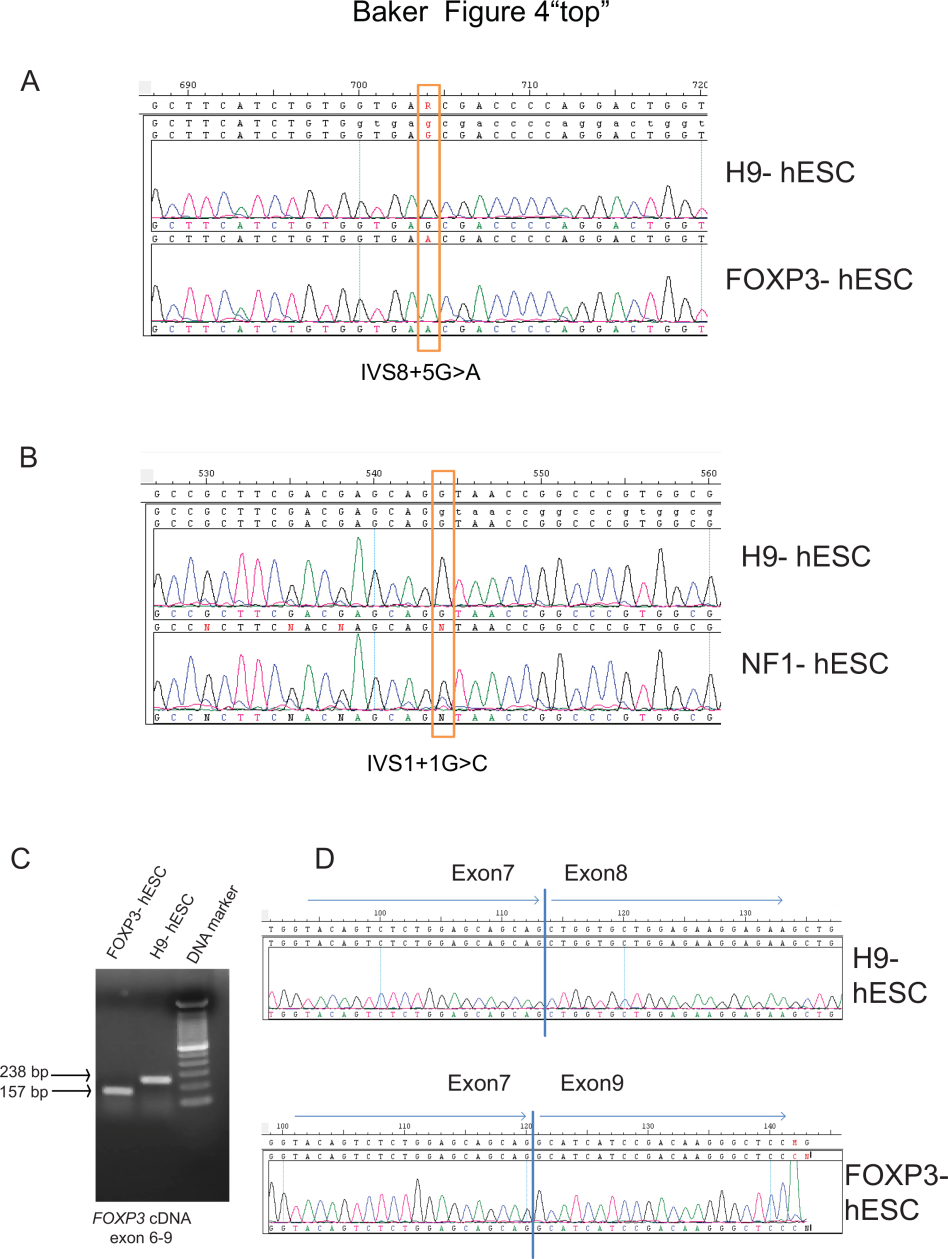
Genetic testing of FOXP3-hESC and NF1-hESC lines. (A and B) PCR analysis and sequencing confirmed *FOXP3* (IVS8+5G>A) mutation in FOXP3-hESC line and *NF1* (IVS1+1G>C, heterozygous of G (black line) and C (blue line)) mutation in NF1-hESC line. H9 hESCs were used as positive control. (C) PCR analysis of exons 6–9 shows that FOXP3-hESC has smaller transcript (157bp) than H9-hESCs (238bp). (D) Sequencing of the PCR fragments revealed the absence of exon 8 in FOXP3-hESC line.

We next sought to test whether the hESC lines contained the lesion associated with the parental mutation. As the FOXP3-hESC line was derived from an embryo containing an X-linked *FOXP3* (IVS8+5G>A) mutation and the NF1-hESC line was derived from embryos heterozygous for a *NF1* (IVS1+1G>C) mutation, we performed PCR with primers that surround these loci, followed by sequencing. Sequencing confirmed that these identical lesions exist in the FOXP3-hESC line (Fig 4A) and the NF1-hESC line (Fig 4B, heterozygous of G (black line) and C (blue line)), confirming that these are indeed hESCs carrying the same mutations as the original PGD embryos. Further, we also sequenced all 12 exons in *FOXP3*, and no other mutation was found (data not shown).

### *FOXP3* (IVS8+5G>A) mutation leads to deletion of a single exon

Most *FOXP3* mutations known to lead to IPEX syndrome are missense and splice-site mutations, affecting protein coding, mRNA stability or splicing [19–21]. To examine the *FOXP3* lesion in more detail, we first tested whether the protein itself was still expressed. Using immunostaining, we find FOXP3 expression in hESCs and no difference in FOXP3 protein expression between H9 and FOXP3-hESC lines (data not shown). We confirmed this finding by flow cytometry [22, 23] (S2 Fig). This suggests that the (IVS8+5G>A) lesion does not lead to absence of protein. We next tested whether the transcript might be altered given the (IVS8+5G>A) lesion. To this end, we amplified *FOXP3* cDNA region from exon 6 to exon 9 and sequenced. Interestingly, we found that the transcript from the FOXP3-hESC line was significantly shorter than that from H9-hESC line (Fig 4C). Sequencing revealed the absence of the entire exon 8 in the FOXP3-hESCs (Fig 4D). To test whether there were other *FOXP3* exon alterations in the FOXP3-hESCs, we also amplified the whole *FOXP3* cDNA from both FOXP3-hESC and from H9-hESC lines, subcloned to pCDH-EF1-T2A-puro expression vector and sequenced. We confirmed the absence of exon 8 and no other exon alteration in the FOXP3-hESCs (data not shown). This strongly suggests that the *FOXP3* IVS8+5G>A lesion causes a splicing defect specifically at this exon, leading to IPEX in these patients. While this is the first molecular analysis of the (IVS8+5G>A) lesion, another *FOXP3* lesion IVS8+4A>G has been reported to cause exon 8 deletion, leading to severe diarrhea, insulin-dependent diabetes, severe eczema and childhood mortality [21].

hESCs carrying disease lesions with known disease phenotypes are tremendous models for drug testing and evaluation of human disorders. This is particularly valuable if the affected cell type can be readily generated from hESCs. IPEX is caused by mutations of FOXP3, a key regulator of T cell homeostasis [3, 4]. Recently, human induced pluripotent stem cells (hiPSCs) were used to efficiently produce T lineage cells [24], suggesting that IPEX may be modeled in these specific cells using the FOXP3-hESC line. Further, it is known that mutations of the *NF1* gene affect normal function of neurofibromin, leading to uncontrolled growth of neural crest cell-derived nerve tissues (e.g., Schwann cells, melanocytes and oligodendrocytes) [8]. Oligodendrocytes have been shown to be efficiently derived from hESC or hiPSC populations [25–27], again making the NF1-hESC line a potential model for functional and drug screening.

In conclusion, we present here the derivation of hESC lines with lesions in *FOXP3* and *NF1*. We demonstrate high efficiency of derivation with 2 lines derived from 6 surviving embryos (50% for *FOXP3* and 25% for *NF1*). Overall, our derivation efficiency from cultured live embryos was approximately 30%. The two lines will be available to the scientific community for the future study.

## Supporting Information

S1 Fig Laser assisted isolation of inner cell mass from blastocyst. (A): Day 5 blastocyst from PGD. (B): Blastocyst being prepared for dissection. (C): The ICM with attached polar trophectodermal cells drawn into the biopsy micropipette. (D): The isolated ICM was then plated on feeder cells.
(TIF)

S2 Fig qRT-PCR analysis of pluripotency marker. qRT-PCR analysis for *OCT4*, *SOX2* and *NANOG* expression in H9-hESC, FOXP3-hESC and NF1-hESC lines. The levels of the transcripts were normalized to *GAPDH*. Data are presented as the mean ± SEM.
(EPS)

S3 Fig qRT-PCR analysis of lineage markers. qRT-PCR analysis for markers of multiple lineages, including endoderm (SOX17and GATA4), mesoderm (*BRACHYURY*), ectoderm (*NESTIN*, *PAX6*) and trophectoderm (CDX2 and *CGA*) in FOXP3-hESC line differentiated into embryoid bodies at day 10. The levels of the transcripts were normalized to *GAPDH*. Data are presented as the mean ± SEM. *, p<0.05; **, p<0.01; ***, p<0.005 (two-tailed *t* test relative to undifferentiated cells).
(EPS)

S4 Fig qRT-PCR analysis of lineage markers. qRT-PCR analysis for markers of multiple lineages, including endoderm (SOX17and GATA4), mesoderm (*BRACHYURY*), ectoderm (*NESTIN*, *PAX6* and *OTX2*) and trophectoderm (CDX2 and *CGA*) in NF1-hESC line differentiated into embryoid bodies at day 10. The levels of the transcripts were normalized to *GAPDH*. Data are presented as the mean ± SEM. *, p<0.05; **, p<0.01; ***, p<0.005 (two-tailed *t* test relative to undifferentiated cells).
(EPS)

S5 Fig FOXP3 expression in H9-hESCs and FOXP3-hESCs. Flow cytometry analysis of FOXP3 expression in K562 cells, MCF7 cells, H9-hESCs and FOXP3-hESCs. Rat IgG2a kappa Isotype Control PE were used as negative control.
(EPS)

S1 File Supplementary Information. (DOCX)
