# Supplementary figures and images for: Human Embryonic Stem Cell Lines with Lesions in FOXP3 and NF1
Source: PLoS One. 2016 Mar 18;11(3):e0151836. doi: 10.1371/journal.pone.0151836 (PMC4798423; doi:10.1371/journal.pone.0151836)

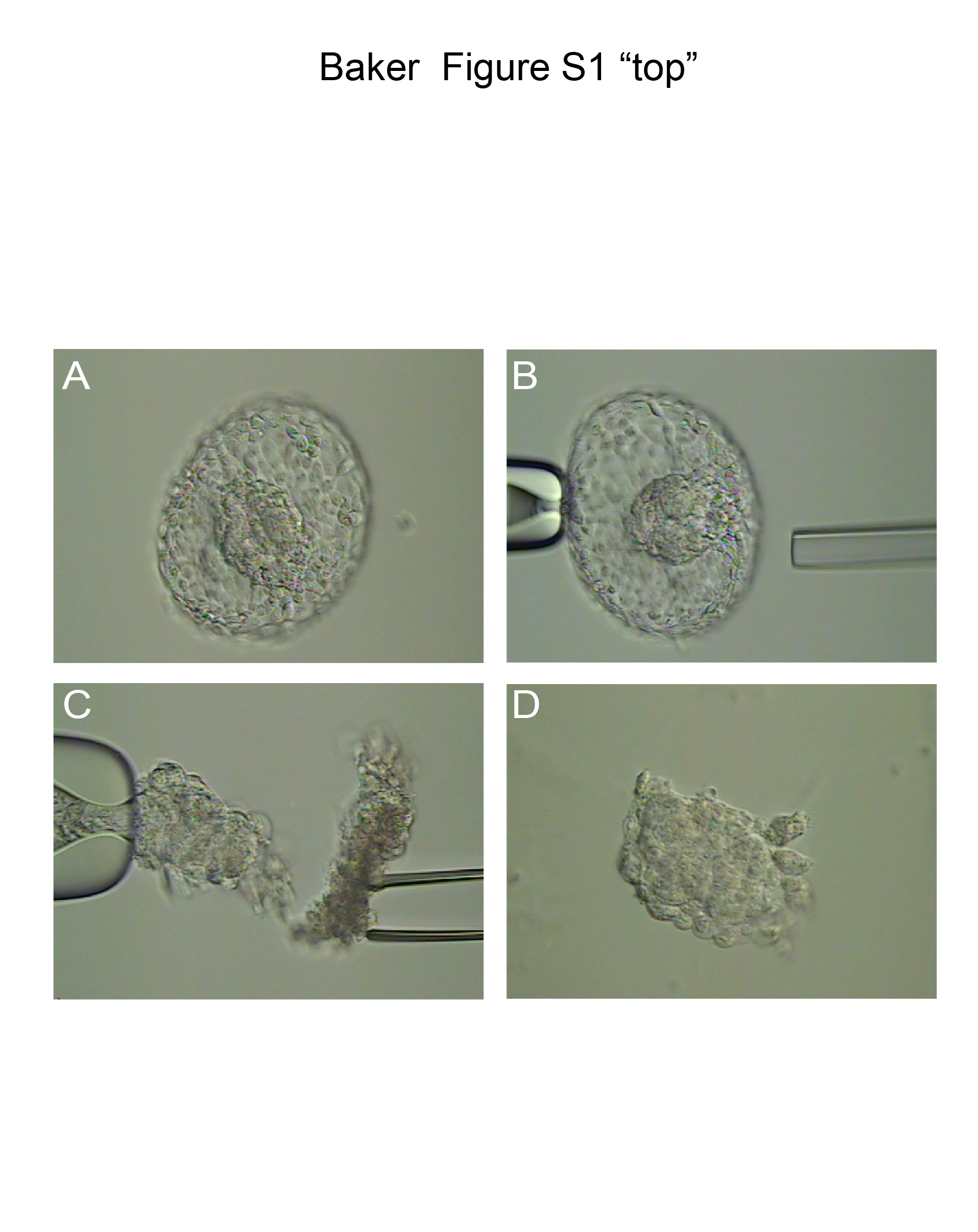

Supplement: S1 Fig — (A): Day 5 blastocyst from PGD. (B): Blastocyst being prepared for dissection. (C): The ICM with attached polar trophectodermal cells drawn into the biopsy micropipette. (D): The isolated ICM was then plated on feeder cells. (TIF) [file pone.0151836.s001.tif]
