# Supplementary material for: Human Embryonic Stem Cell Lines with Lesions in FOXP3 and NF1
Source: PLoS One. 2016 Mar 18;11(3):e0151836. doi: 10.1371/journal.pone.0151836 (PMC4798423; doi:10.1371/journal.pone.0151836)
Supplement: S1 File — (DOCX) [file pone.0151836.s006.docx]

**Supplementary Experimental Procedures**

**Analysis of mRNA**

RNA was extracted by using RNeasy Plus Mini Kit (Qiagen, Duesseldorf, Germany) and then used for reverse transcription with Oligo dT using SuperScript III First-Strand cDNA synthesis kit (Invitrogen). Quantitative RT-PCR (qRT-PCR) reactions were run using 50 nM forward and reverse primers with iQ SYBR Green Supermix (Bio-Rad, Hercules, CA). Real-time PCR was performed using the Bio-Rad iCycler: 94°C for 5 min followed by 40 cycles consisting of denaturation (95°C, 30 s), annealing (55°C, 30 s), and extension (72°C, 30 s), with a final incubation at 72°C for 10 min. Relative mRNA level was normalized to of the levels of *GAPDH*. PCR was performed in triplicate for each sample, and 3 independent experiments were carried out. Primers for qRT-PCR are listed in S2 Table.

**Embryoid body differentiation of hESCs**

hESCs were dissociated to small colonies by collagenase. Embryoid body formation was induced by seeding ~5X10^5 cells in each well of ultralow attachment 6-well plates (Corning) in hESCs culture medium without bFGF.

**Flow Cytometry Analysis**

FOXP3 expression in cell lines was analyzed by flow cytometry using Anti-Human FOXP3 PE Staining Set (72-5776-40, eBioscience, Inc., San Diago, CA). Cells were fixed and permeabilized using fixing and staining buffers and subsequently stained with Anti-Human FOXP3 PE. Cells in a separate tube treated in parallel with Rat IgG2a kappa Isotype Control PE was used as a negative control. The cells were examined by fluorescence-activated cell sorting (FACS) using a flow cytometer (FACSCAN, BD Bioscience) in the Stanford Shared FACS Facility. Data were analyzed using the Flowjo software (Tree Star, Inc., Ashland, Oregon). The procedure was performed according to the manufacture’s instruction.

**Supplementary table information**

Table A. PCR primer sequences

|  | Forward primer (5’-3’) | Reverse primer (5’-3’) |
| --- | --- | --- |
| Primers for amplifying *FOXP3* exons |  |  |
| Exon 1 | ggagaaacacagagagagagaaaaa | ctacctccctgccatctcct |
| Exon 2 | ggctcaggtggtcgagtatc | cccagtgccacagtaaaggt |
| Exon 3 and 4 | gggcttgcagtgcagagtat | ctgctcacagccaaggatct |
| Exon 5 | gctcaaaatgagaggccttg | gggacagagggtgtcagg |
| Exon 6 | aggaaggacaggtcagtgga | accctagacctctccccaca |
| Exon 7 | cctccccattcagagcatt | cccccagcagtctgagtc |
| Exon 8 | ggtggagggtaagggatagg | tgaggttaggttccctgcac |
| Exon 9 | aaatagaaagtaaaaagtgggaagttt | ctcctttgcaccctccac |
| Exon 10 | ggacagggagctagctaggag | ggaatggaggaacccactct |
| Exon 11 and 12 | tagcccctctaaaccccaag | cacatccagggcctatcatc |
| Primers for amplifying *FOXP3* cDNA containing exon 6-7-8 | gtggcccggatgtgagaag | tgggcctgcatggcactca |
| Primers for amplifying *FOXP3* cDNA containing exon 6-7-9 | gtggcccggatgtgagaag | ggagcccttgtcggatgatg |
| Primers for amplifying the whole *FOXP3* cDNA | ctagcgaattcgccaccatgcccaaccccaggcct | ccctcagcggccgcggggccaggtgtag  ggttggaacac |
| Primers for region surrounding IVS1+1G>C in *NF1* gene | ctgcactccacagaccctct | tcccctcacctactctgtcc |

Table B. qRT-PCR primer sequences

| Gene | Forward primer (5’-3’) | Reverse primer (5’-3’) |
| --- | --- | --- |
| *OCT4* | GTGGAGGAAGCTGACAACAA | ATTCTCCAGGTTGCCTCTCA |
| *NANOG* | ATGCCTCACACGGAGACTGT | AGGGCTGTCCTGAATAAGCA |
| *SOX2* | ATGGGTTCGGTGGTCAAGT | GGAGGAAGAGGTAACCACAGG |
| *GATA4* | GGAAGCCCAAGAACCTGAAT | CTGGAGTTGCTGGAAGCAC |
| *SOX17* | ACGCCGAGTTGAGCAAGA | TCTGCCTCCTCCACGAAG |
| *NESTIN* | TGCGGGCTACTGAAAAGTTC | TGTAGGCCCTGTTTCTCCTG |
| *PAX6* | TCACCATGGCAAATAACCTG | CAGCATGCAGGAGTATGAGG |
| *BRACHYURY* | ACAGCGCATGATCACCAG | TTTGCAAATGGATTGTACTTAATTTT |
| *OTX2* | GCAGAGGTCCTATCCCATGA | CTGGGTGGAAAGAGAAGCTG |
| *CDX2* | CCGAACAGGGACTTGTTTAGAG | CTCTGGCTTGGATGTTACACAG |
| *CGA* | CTTTCTGCATGTTCTCCATTC | GTGGACTCTGAGGTGACGT |
